# Efficacy and safety of low-dose rosuvastatin/ezetimibe for dyslipidemia in patients with rheumatoid arthritis or osteoarthritis

**DOI:** 10.1097/MD.0000000000043133

**Published:** 2025-07-04

**Authors:** Seong-Hyeok Bak, Kyung-Ann Lee, Sung Soo Kim, Sang-Hyon Kim, Seung-Jae Hong, Hyun-Sook Kim

**Affiliations:** a Division of Rheumatology, Department of Internal Medicine, Soonchunhyang University Seoul Hospital, Seoul, Republic of Korea; b Division of Rheumatology, Department of Internal Medicine, Gangneung Asan Hospital, Ulsan University College of Medicine, Gangneung, Republic of Korea; c Division of Rheumatology, Department of Internal Medicine, Keimyung University Dongsan Medical Center, Daegu, Republic of Korea; d Division of Rheumatology, Department of Internal Medicine, Kyung Hee University Medical Center, Seoul, Republic of Korea.

**Keywords:** dyslipidemia, ezetimibe, osteoarthritis, rheumatoid arthritis, rosuvastatin

## Abstract

**Background:**

Dyslipidemia is an important condition in patients with rheumatoid arthritis (RA). This study compared the effectiveness and safety of a low-dose rosuvastatin/ezetimibe formulation for dyslipidemia in RA and osteoarthritis (OA).

**Methods:**

This multicenter, open-label, clinical trial enrolled patients >19 years of age with RA and or hand/knee OA who met the prescribing indications for rosuvastatin/ezetimibe in primary hypercholesterolemia or mixed dyslipidemia, and the Korean insurance criteria. All the patients received rosuvastatin 5 mg/ezetimibe 10 mg daily. The primary endpoint was a low-density lipoprotein cholesterol (LDL-C) reduction ≥ 50% from baseline at 12 weeks.

**Results:**

The study recruited 162 patients with RA and 119 with OA, of whom 143 RA and 107 OA patients completed the follow-up and were included in the final analysis. Compared to the OA group, the RA group was older, had more females, had a lower body mass index, and a higher C-reactive protein level. The primary endpoint of an LDL-C reduction ≥ 50% from baseline was achieved in 79.7% of the RA group and 70.1% of the OA group (*P *= .1086). No significant differences were found in the safety endpoints. In the univariate linear regression analysis, baseline LDL-C levels were associated with absolute LDL-C reduction. This association remained significant in the multivariate analysis. The Disease Activity Score-28 for RA with erythrocyte sedimentation rate, C-reactive protein level, age, body mass index, current smoking, hypertension, diabetes, and daily glucocorticoid dose, were not significantly associated.

**Conclusion:**

The efficacy and safety of low-dose rosuvastatin/ezetimibe in reducing LDL-C levels were similar between the RA and OA groups. Baseline LDL-C level was the sole independent factor associated with LDL-C reduction, irrespective of inflammatory status.

## 1. Introduction

Patients with rheumatoid arthritis (RA) have a higher cardiovascular disease (CVD) risk than the general population. A previous meta-analysis showed that the risk of coronary artery disease mortality was 50% higher in RA patients than in the general population.^[1]^ Therefore, dyslipidemia is actively managed as a traditional risk factor for atherosclerotic cardiovascular disease (ASCVD) when treating RA. In the general population, high total cholesterol (TC) and low-density lipoprotein cholesterol (LDL-C) mean a high CVD risk.^[2]^ In RA patients, findings showing a nonlinear relationship between LDL-C and CVD risk have been reported, which came to be described as the lipid paradox.^[3]^ Liao et al^[4]^ reported that subjects at the highest risk for major adverse cardiovascular events had the lowest and highest LDL-C levels. Navarro-Millán et al^[5]^ reported no clear association between LDL-C level and the risk of myocardial infarction stroke or death. Inflammation appears to be inversely associated with lipid levels in RA. In studies of patients with RA, treatment with biologics or Janus kinase inhibitors has been reported to increase lipid levels in those who are not taking statins.^[6]^ Inflammation may modulate the impact of lipid measures on CVD and appears to contribute to increased CVD risk in RA. However, it remains uncertain which inflammation confounds the association between cholesterol and CVD.^[3,7]^ Still, undertreatment of traditional risk factors in patients with RA has been suggested to contribute to CVD risk.^[8]^

Statins are considered the first-line treatment due to their efficacy and good safety profile in the general population. It appears to have similar efficacy in patients with RA.^[2]^ Statins can cause hepatic dysfunction and muscle-related adverse events (e.g., rhabdomyolysis and necrotizing myopathy), and are associated with new-onset diabetes mellitus (DM), in a dose-dependent manner.^[9]^ In a large retrospective cohort study, statin use was associated with disease progression in DM.^[10]^ Adding ezetimibe to statin therapy can be an alternative to increasing the statin dose.^[11]^ In a study of patients with atherosclerotic CVD, rosuvastatin 10 mg with ezetimibe 10 mg had fewer adverse events and intolerance than rosuvastatin 20 mg.^[12]^

RA is a systemic inflammatory autoimmune disease that preferentially affects the joints. RA and ASCVD share inflammatory pathways, including the IL-1, IL-6, and TNF pathways. As in RA, the inflammation of osteoarthritis (OA) involves IL-1, IL-6 and TNF. The concentrations of acute phase reactants are elevated in OA but to a lesser extent than those in RA.^[13]^

As far as we are aware, there is no clear evidence that chronic inflammation reduces the efficacy or increases the side effects of statins and ezetimibe. In a previous retrospective study of patients with and without inflammatory arthritis, statin treatment led to a comparable decrease in lipid levels.^[14]^ We compared the efficacy of a rosuvastatin and ezetimibe combination therapy in a real-world setting – where minimal disease progression is expected – between patients with RA, an inflammatory arthritis, and OA, a non-inflammatory arthritis. We hypothesized that there would be no difference in the efficacy of the statin and ezetimibe combination therapy between RA and OA.

## 2. Methods

### 2.1. Study design and population

This multicenter, open-label, clinical trial enrolled patients with RA or hand or knee OA at 4 referral hospitals in the Republic of Korea between June 2021 and December 2023. Patients with OA, like those with RA, are expected to maintain regular clinical visits and have comparable demographic profiles. Patients with RA were diagnosed with RA according to the 1987 American College of Rheumatology (ACR) or the 2010 ACR/European League Against Rheumatism classification criteria.^[15,16]^ OA patients were diagnosed with OA according to the ACR hand or knee OA classification criteria.^[17]^ The study enrolled patients aged ≥19 years from both groups, who met the prescribing indications for rosuvastatin/ezetimibe in primary hypercholesterolemia or mixed dyslipidemia, and the Korean insurance criteria for rosuvastatin/ezetimibe. These criteria are detailed in Supplementary Tables S1 and S2, Supplemental Digital Content, https://links.lww.com/MD/P299. All patients received a rosuvastatin 5 mg/ezetimibe 10 mg formulation. The patients had no history of statin or fibrate administration or a last dose date >4 weeks earlier. The detailed inclusion and exclusion criteria are provided in Supplementary Tables S1 and S2, Supplemental Digital Content, https://links.lww.com/MD/P299. Patients with RA and OA were classified into the RA group. Patients with OA but without coexisting inflammatory arthritis were assigned to the OA group.

This study was conducted in accordance with the Declaration of Helsinki and was approved by the Institutional Review Board of Soonchunhyang University Seoul Hospital (Institutional Review Board Number: 2021-05-005). Written informed consent was obtained from all the participants.

### 2.2. Data collection

At the start of the rosuvastatin/ezetimibe treatment, baseline demographic clinical, and biochemical data were collected. In the RA group, the rheumatoid factor titer, antibodies against cyclic citrullinated peptide antibody (anti-CCP antibodies) titer, disease duration, medication history, disease activity score with the erythrocyte sedimentation rate (DAS28-ESR), and whether the diagnosis was made according to the classification criteria were obtained. In the OA group, the diagnosis was confirmed according to the classification criteria. Medications, accompanying systemic diseases were collected in both groups. Drinking and smoking histories were assessed using a standardized questionnaire.

### 2.3. Endpoints

For those who agreed, primary and secondary endpoints were analyzed by accessing medical records and laboratory tests related to treatment at baseline and the 4 ± 2, and 12 ± 4 week visits. The primary endpoint was a LDL-C reduction ≥ 50% from baseline at 12 weeks. The secondary endpoints were the rate of LDL-C < 70 mg/dL at 12 weeks in each group, the amount (mg/dL) and rate (%) of change in TC, triglyceride (TG), LDL-C, high-density lipoprotein cholesterol (HDL-C), and non-HDL-C compared to baseline at 12 weeks in each group, and the rate (%) of adverse events major vascular events, death, new-onset DM, rhabdomyolysis, significant liver enzyme elevation and increased creatinine level after rosuvastatin/ezetimibe administration in each group. A major vascular event was defined as a diagnosis of myocardial infarction or stroke. Significant liver enzyme elevation was defined as an increase in AST or ALT to >3 times the baseline value and above the upper limit of the normal range. An increased creatinine level was defined as an increase in serum creatinine of ≥0.3 mg/dL or ≥50% from baseline.

### 2.4. Statistical analysis

Statistical analyses were performed using SPSS (ver.22.0; SPSS, Chicago, IL). Continuous variables were expressed as numbers, means, standard deviations, medians, and ranges, while categorical variables were present as frequency and percentage. Unless otherwise specified, all tests were performed on the principle of 2-tailed testing at a significance level of 5%. Intergroup comparisons were performed using the Mann–Whitney *U* test for continuous variables, and the chi-square or Fisher exact test for categorical data. *P* values < 0.05 were considered statistically significant. In the absence of prior data, we conservatively estimated the sample size assuming a clinically meaningful difference of 20% between groups. With a 2-sided *α* of 0.05 and 80% power, and allowing for a 5% dropout rate, the final sample size was calculated to be 102 participants per group (total N = 204).

## 3. Results

### 3.1. Patient characteristics

**Figure 1.**
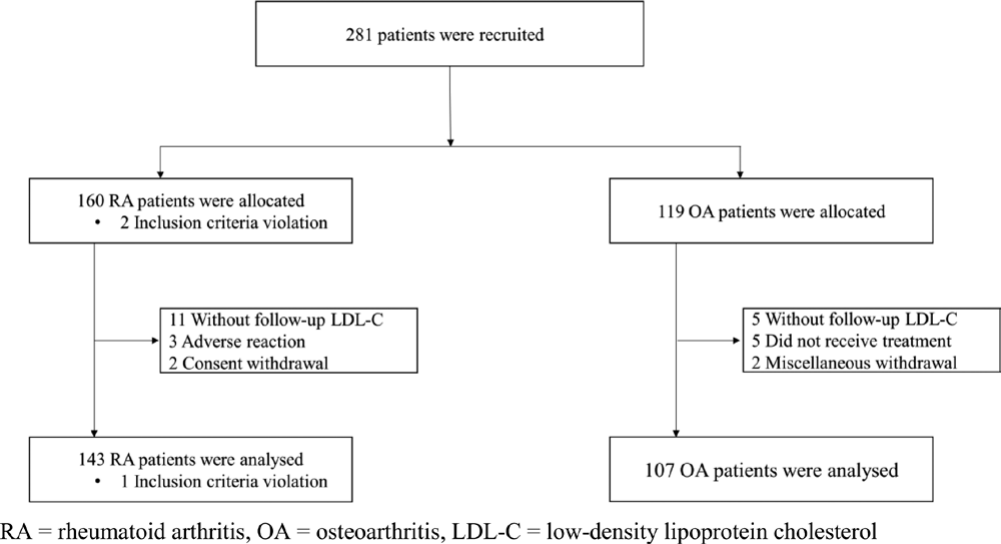
Trial flow. RA = rheumatoid arthritis, OA = osteoarthritis, LDL-C = low-density lipoprotein cholesterol.

Between June 2021 and December 2023, 281 patients were recruited: 162 with RA and 119 with OA. After exclusion due to protocol violations, loss to follow-up, adverse events, or consent withdrawal, 143 RA and 107 OA patients were included in the final analysis (Fig. 1).

Table 1 summarizes the baseline demographics and the clinical and biochemical characteristics of the patients. RA patients were slightly older than OA patients (median 60 vs 58.5 yr, *P* = .0226), and the proportion of male patients was lower in the RA group (23.8% vs 36.5%, *P* = .035). The body mass index (BMI) was lower in RA patients (23.63 vs 24.7 kg/m², *P* = .0084). In lipid profiles, HDL-C was significantly higher in RA (59 vs 52 mg/dL, *P* < .001), while LDL-C, TC, and TG levels were similar. As expected, RA patients had higher use of glucocorticoids (78.3% vs 19.6%, *P* < .001), methotrexate (61.5%), and biologic disease-modifying anti-rheumatic drugs (bDMARDs) (21%). C-reactive protein (CRP) levels were higher in RA patients (0.15 vs 0.1 mg/dL, *P* = .0036), and the median DAS28-ESR score was 2.85.

**Table 1:** Baseline demographic, clinical, and biochemical characteristics.

	RA (n = 143) n(%)	OA (n = 107) n(%)	*P*
Age, median (IQR), yr	60 (54–67)	58.5 (49,65)	.0226*
Male sex	33 (23.77)	39 (36.45)	.035†
Body mass index, median (IQR), (kg/m^2^)	23.63 (22–25.8)	24.7 (22.66–26.95)	.0084*
Systolic blood pressure, median (IQR), (mm Hg)	128 (120–137)	128 (120–138)	.4784*
Diastolic blood pressure, median (IQR), (mm Hg)	77 (70–82)	80 (71–82.5)	.27*
Vascular risk factors			
Hypertension	55 (38.46)	39 (35.51)	.7941†
Diabetes mellitus	6 (4.20)	6 (5.61)	.8181†
Current smoking	25 (17.48)	13 (12.15)	.348†
Lipid profiles (mg/dL)			
LDL-C, median (IQR)	162 (148.75–177)	169 (154.5–181)	.0548*
TC, median (IQR)	251 (234–274)	253 (231–272)	.9776*
TG, median (IQR)	137 (105–183)	148 (113–190)	.1656*
HDL-C, median (IQR)	59 (50–70)	52 (46–63.5)	<.001*
Glucose, median (IQR), (mg/dL)	97 (91–104)	102 (96–109)	<.001*
eGFR, mean ± SD, (mL/min)	88.42 ± 19.89	91.26 ± 17.47	.2319‡
ESR, median (IQR), (mm/h)	21 (6–39)	19 (4–32)	.1174*
CRP, median (IQR), (mg/dL)	0.15 (0.08–0.36)	0.1 (0.06–0.19)	.0036*
AST, median (IQR), (U/L)	23 (19–28)	25 (19–30)	.1953*
ALT, median (IQR), (U/L)	19 (13–25)	21 (16–31)	.0033*
DAS28-ESR, median (IQR)	2.85 (2.26–3.78)		
Current medication			
Glucocorticoid	112 (78.32)	21 (19.62)	<.001†
Methotrexate	88 (61.53)		
bDMARDs	30 (20.97)		

ALT = alanine transaminase, AST = aspartate transaminase, bDMARDs = biologic disease-modifying anti-rheumatic drugs, CRP = c-reactive protein, DAS28 = disease activity score-28, eGFR = estimated glomerular filtration rate, ESR = erythrocyte sedimentation rate, HDL-C = high-density lipoprotein, IQR = intra quartile range, LDL-C = low-density lipoprotein cholesterol, OA = osteoarthritis, RA = rheumatoid arthritis, TC = total cholesterol, TG = triglyceride.

* Mann–Whitney. †Chi-square. ‡T test.

### 3.2. Efficacy outcomes

The proportion of patients achieving the primary endpoint – a ≥50% reduction in LDL-C – was higher in the RA group than in the OA group (79.7% vs 70.1%), although the difference was not statistically significant (*P* = .1086) (Table 2). Secondary endpoints, including LDL-C < 70 mg/dL and composite goals, were more frequently achieved in the RA group, but without statistical significance. Both groups showed similar absolute and percentage reductions in LDL-C levels. Multiple lipid goal achievement was significantly higher in the RA group (49.7% vs 30.8%, *P* = .0043).

**Table 2:** Efficacy outcomes.

Endpoints	RA (n = 143) n(%)	OA (n = 107) n(%)	*P* value
Primary endpoint			
LDL-C reduction ≥ 50%	114 (79.72)	75 (70.09)	.1086†
Secondary endpoints			
LDL-C < 70 mg/dL	85 (59.44)	52 (48.6)	.115†
LDL-C reduction ≥ 50% or LDL-C < 70 mg/dL	114 (79.72)	76 (71.03)	.1491†
Absolute LDL-C reduction, median (IQR), (mg/dL)	97 (80, 112)	97 (68, 114)	.4009*
LDL-C reduction percentage, median (IQR), (%)	60.77 (53.15, 66.7)	59.7 (43.6, 67.69)	.2512*
Multiple lipid goal achievement	71 (49.65)	33 (30.84)	.0043†

LDL-C = low-density lipoprotein cholesterol, OA = osteoarthritis, RA = rheumatoid arthritis.

* Mann–Whitney. †Chi-square.

There were no significant differences between the RA and OA groups in the incidence of adverse events, including deaths (0% vs 0%), new-onset DM (0.6% vs 0.9%), rhabdomyolysis (0% vs 0%), significant liver enzyme elevation (1.3% vs 0 %) and increased creatinine level (0.69% vs 0%) (Table 2).

### 3.3. Factors associated with achieving the primary endpoint in patients with RA

Within the RA group, we compared the groups that achieved the primary endpoint with those that did not. There were no significant differences in age, sex distribution, BMI, blood pressure, or vascular risk factors including hypertension, diabetes, and current smoking status. The baseline lipid profiles, renal function, inflammatory markers (ESR and CRP), disease activity (DAS28-ESR), and treatment were also comparable (Table 3).

**Table 3:** Characteristics of patients with RA according to a ≥50% reduction in LDL-C.

	LDL ≥ 50% reduction (n = 114)	LDL < 50% reduction (n = 29)	*P* value
Age, mean ± SD, (yr)	59.89 ± 9.15	62.69 ± 8.69	.1319‡
Male sex	30 (26.32)	3 (10.34)	.1151†
Body mass index, median (IQR), (kg/m^2^)	23.66 (22.01, 26.07)	23.78 (22, 24.96)	.8942*
Systolic blood pressure, median (IQR), (mm Hg)	127 (119.25, 136.75)	129 (122, 137)	.2358*
Diastolic blood pressure, median (IQR), (mm Hg)	76.5 (70, 82)	78 (70, 81)	.9559*
Vascular risk factors			
Hypertension	41 (35.96)	14 (48.28)	.3159†
DM	6 (5.26)	0 (0)	.3466§
Current smoker	20 (17.54)	4 (13.79)	.7843§
Baseline lipid profiles (mg/dL)			
LDL-C, median (IQR)	164 (149, 180)	161 (146, 166)	.1321*
TC, mean ± SD	255.78 ± 31.54	249.84 ± 27.39	.4126‡
TG, median (IQR)	138 (108, 177.25)	133 (91, 196)	.8625*
HDL-C, median (IQR)	61 (50, 70)	57 (51, 65)	.792*
eGFR, mean ± SD, (mL/min)	87.86 ± 20.93	91.56 ± 14.51	.2714‡
ESR, median (IQR), (mm/h)	21 (5, 42)	21 (7, 33)	.9636*
CRP, median (IQR), (mg/dL)	0.17 (0.09, 0.41)	0.1 (0.05, 0.2)	.1676*
DAS28-ESR, mean ± SD	2.97 ± 1.23	3.02 ± 1.19	.8471‡
Current medication			
GC	93 (81.58)	19 (65.52)	.1048†
Daily GC dose, median (IQR), (mg/d)	5 (2.5, 5)	5 (3, 5)	.4356*
bDMARDs	24 (21.05)	6 (20.69)	>.99†
Methotrexate	67 (58.77)	20 (68.97)	.4289†

bDMARDs = biologic disease-modifying anti-rheumatic drugs, CRP = c-reactive protein, DAS28 = disease activity score-28, DM = diabetes mellitus, eGFR = estimated glomerular filtration rate, ESR = erythrocyte sedimentation rate, GC = glucocorticoid, HDL-C = high-density lipoprotein, IQR = inter quartile range, LDL-C = low-density lipoprotein cholesterol, RA = rheumatoid arthritis, TC = total cholesterol, TG = triglyceride.

*Mann–Whitney.

*Chi-square. ‡*T* test. §Fisher exact.

In univariate linear regression analysis, baseline LDL-C level was associated with absolute LDL-C reduction (*β* = 0.7009, 0.5113–0.8905, *P *< .001) (Table 4). This association remained significant in multivariate analysis (*β* = 0.744, 0.529–0.959, *P *< .001). Other factors, including sex, age, BMI, current smoking, hypertension, diabetes, CRP level, DAS28-ESR, and daily glucocorticoid dose, were not significantly associated with LDL-C reduction.

**Table 4:** Linear regression analysis of factors associated with absolute LDL-C reduction in patients with RA.

	Univariate				Multivariate			
	*β*	Lower	Upper	*P* value	*β*	Lower	Upper	*P* value
Sex (male)	8.117	−4.5143	20.7484	.206	9.5368	−2.4974	21.571	.1191
Age	−0.135	−0.7277	0.4578	.6533				
BMI	0.8796	−0.6963	2.4555	.2717				
Current smoking	2.6434	−11.6671	16.9539	.7155				
Hypertension	−3.5902	−14.5884	7.408	.5197				
DM	17.0148	−9.4378	43.4674	.2056	21.4289	−3.7154	46.5732	.094
Baseline LDL-C	0.7009	0.5113	0.8905	<.001	0.744	0.529	0.959	<.001
CRP	−0.5062	−11.5041	10.4917	.9276				
DAS28-ESR	0.6711	−3.8038	5.146	.7673				
Daily GC dose	−0.8313	−3.5072	1.8445	.5393				

BMI = body mass index, CRP = c-reactive protein, DAS28 = disease activity score-28, DM = diabetes mellitus, ESR = erythrocyte sedimentation rate, GC = glucocorticoid, LDL-C = low-density lipoprotein cholesterol, RA = rheumatoid arthritis.

## 4. Discussion

In this study, we designated patients with OA as the control group for patients with RA. Patients with OA are expected to be demographically similar to RA patients.^[18,19]^ By including OA patients, we established a control group without inflammatory arthritis. Previous studies have shown that OA severity is not associated with systemic inflammation,^[13,20,21]^ and traditional ASCVD risk factors – except for BMI – did not differ significantly between the groups (Table 1). Therefore, we believe that this control group with consistently lower systemic inflammation than RA patients was appropriate.

In previous large retrospective studies, statin therapy in RA patients led to a reduction in cardiovascular events comparable to that observed in the general or OA populations. As discussed in the Introduction, there is no definitive evidence that the efficacy of statins is diminished by systemic inflammation.^[2,19]^ Accordingly, we hypothesized that the efficacy of rosuvastatin/ezetimibe would not differ between patients with RA and OA. The rate of achieving the primary endpoint was not significantly different between the 2 groups (Table 2).

Within the RA group, there were no significant differences in ESR, CRP, DAS28, glucocorticoid dose, use of bDMARDs, or methotrexate between those who achieved the primary endpoint and those who did not. Similarly, traditional factors that could influence systemic inflammation did not differ significantly (Table 3). In linear regression analysis using absolute LDL-C reduction as the dependent variable, the baseline LDL-C level was the only factor significantly associated with LDL-C reduction (Table 4).

In addition to statins and ezetimibe, lipid-lowering agents such as fibrates and PCSK9 inhibitors are also available. However, fibrates are not routinely added to statin therapy, as current evidence does not support their widespread use. Moreover, their efficacy has only been demonstrated in patients with elevated TGs.^[22–24]^ PCSK9 inhibitors lack clear superiority in efficacy and safety over statins and are considerably more expensive. These 2 drugs are expected to be used as alternative options or in specific subgroups.

Despite the passage of time, the underuse of treatment for traditional CVD risk factors in patients with RA continues to be reported.^[25,26]^ This trend appears to extend beyond certain countries.^[6]^ It has been reported that, with adequate RA treatment, most of the current increased CVD risk is mediated through traditional risk factors.^[8]^ Therefore, we believe that there is a strong rationale for active use of lipid-lowering agents in patients with RA.

Because lipid-lowering therapy is typically initiated with low-dose agents in clinical practice, this study aimed to evaluate the efficacy and safety of this low-dose formulation. Elevation of lipid levels during the continued use of first-line RA therapies has been reported in a previous study.^[6]^ Taken together with prior research, we cautiously propose the following: in patients with RA who are not receiving lipid-lowering therapy, it may be appropriate to initiate statin or ezetimibe therapy while concurrently managing RA. After achieving better disease activity, lipid profiles can be reevaluated to determine whether to continue, discontinue, or escalate therapy.

This study had several limitations. First, the disease activity in the RA group was generally low, which may have limited generalizability. However, even considering the low disease activity of our study population, appropriate treatment with conventional synthetic DMARDs or bDMARDs is expected to lower inflammation levels, and the efficacy and safety of lipid-lowering agents are likely to be maintained as observed in our study. Second, an open-label design was selected. However, based on previous similar studies reporting few adverse events, we anticipated that this design might help detect adverse reactions via the nocebo effect.^[27,28]^ Moreover, the primary endpoint was objective laboratory values, which minimize subjectivity. Third, we did not sufficiently evaluate adherence. Non-adherence to the medication regimen may have led to an underestimation of the true efficacy of rosuvastatin/ezetimibe. Finally, the inflammatory markers collected in this study may not have been sufficient to fully elucidate the relationship between inflammation and lipid levels. Nevertheless, since ESR and CRP are standard markers used to assess RA disease activity in clinical practice, we believe they were adequate to test the study hypothesis.

## 5. Conclusion

Our study demonstrated that low-dose rosuvastatin/ezetimibe was equally effective and safe for patients with RA and OA. These findings support the initiation of lipid-lowering therapy regardless of the level of RA-driven systemic inflammation.

## Author contributions

**Conceptualization:** Hyun-Sook Kim.

**Data curation:** Seong-Hyeok Bak, Kyung-Ann Lee, Sung Soo Kim, Sang-Hyon Kim, Seung-Jae Hong.

**Formal analysis:** Seong-Hyeok Bak, Kyung-Ann Lee.

**Funding acquisition:** Hyun-Sook Kim.

**Investigation:** Sung Soo Kim, Sang-Hyon Kim, Seung-Jae Hong, Hyun-Sook Kim.

**Methodology:** Hyun-Sook Kim.

**Project administration:** Hyun-Sook Kim.

**Resources:** Hyun-Sook Kim.

**Software:** Kyung-Ann Lee.

**Supervision:** Hyun-Sook Kim.

**Validation:** Seong-Hyeok Bak, Kyung-Ann Lee.

**Visualization:** Kyung-Ann Lee.

**Writing – original draft:** Seong-Hyeok Bak.

**Writing – review & editing:** Kyung-Ann Lee.

## Supplementary Material


