# Supplementary material for: Efficacy and safety of low-dose rosuvastatin/ezetimibe for dyslipidemia in patients with rheumatoid arthritis or osteoarthritis
Source: Medicine (Baltimore). 2025 Jul 4;104(27):e43133. doi: 10.1097/MD.0000000000043133 (PMC12237359; doi:10.1097/MD.0000000000043133)
Supplement: Supplementary file 1 [file medi-104-e43133-s001.docx]

**Supplementary Table 1**. Inclusion and exclusion criteria

| Inclusion criteria |
| --- |
| 1. Age ≥ 19  2. RA diagnosed according to the 1987 ACR or the 2010 ACR/EULAR classification criteria (RA group) or OA diagnosed according to the ACR hand/knee OA classification criteria (OA group)  3. Meets the treatment indications for primary hypercholesterolemia or mixed dyslipidemia, or meets the Korean insurance criteria* for rosuvastatin/ezetimibe administration  4. Subjects with no history of statin or fibrate administration, or last dose date more than 4 weeks  5. Informed consent provided |
| Exclusion criteria |
| 1. Refusal to agree to participate  2. Subjects who received statin or fibrate within 4 weeks  3. Subjects with hypersensitivity reactions to rosuvastatin or ezetimibe  4. Subjects with active myopathy (includes rhabdomyolysis)  5. Patients with active liver disease or serum transaminase ≥ 2 times ULN or Severe kidney failure (creatinine clearance < 30mL/min) or uncontrolled DM(blood glucose > 400 mg/dL)  6. Uncontrolled hypertension (BP ≥ 160/100 mmHg in screening)  7. Pregnant or potentially pregnant women and lactating women  8. Have a history or family history of hereditary myopathy  9. History of myopathy related to statins(HMG-CoA reductase inhibitor) or fibrates  10. Subjects who have received other investigational drugs or medical devices within 30 days before screening  11. History of drug or alcohol abuse within 1 year prior before screening  12. Malignancy within 5 years before to screening |

RA = rheumatoid arthritis, OA = osteoarthritis, ACR = American College of Rheumatology, EULAR = European League Against Rheumatism, ULN = upper limit normal, DM = diabetes mellitus

*See Supplementary Table 3.

**Supplementary Table 2**. Abbreviated National Health Insurance Service for rosuvastatin/ezetimibe formulation indication

| (A) LDL-C dyslipidemia |
| --- |
| 1Indication  a. 0~1 risk factor : LDL-C ≥ 160 mg/dL  b. 2 ≥ risk factor: LDL-C ≥ 130 mg/dL  c. If the patient has coronary artery disease or peripheral artery disease, abdominal aortic aneurysm, symptomatic carotid artery disease, serum LDL-C ≥ 100 mg/dL with DM  d. Acute coronary syndrome patients : LDL-C ≥ 70 mg/dL  2. Applicable drugs: One of HMG-CoA reductase inhibitor, bile acid sequetrant, or fibrates |
| (B) Hypertriglyceridemia |
| 1. Indication  a. TG ≥ 500 mg/dL  b. If the patient has a risk factor* or diabetes mellitus : TG ≥200 mg/dL  2.Applicable drugs: One of fibrates and niacin |
| (C) LDL-C dyslipidemia with hypertriglyceridemia |
| 1.Indication  a. According to (A) or (B)  2. Applicable drugs: One drug each of (A) and (B) |
| * Risk factors |
| 1. Smoking  2. Hypertension (BP ≥ 140/90 mmHg or taking hypertension drugs)  3. HDL-C < 40 mg/dL  4. Family history of early onset coronary artery disease  # HDL-C ≥ 60 mg/dL is considered a protective factor and subtracts one from the total number of risk factors |

DM = diabetes mellitus, LDL-C = low-density lipoprotein cholesterol, TG = triglyceride, HDL-C = high-density lipoprotein cholesterol
